# Putative Biomarkers in Tears for Diabetic Retinopathy Diagnosis

**DOI:** 10.3389/fmed.2022.873483

**Published:** 2022-05-25

**Authors:** Madania Amorim, Beatriz Martins, Francisco Caramelo, Conceição Gonçalves, Grimalde Trindade, Jorge Simão, Patrícia Barreto, Inês Marques, Ermelindo Carreira Leal, Eugénia Carvalho, Flávio Reis, Teresa Ribeiro-Rodrigues, Henrique Girão, Paulo Rodrigues-Santos, Cláudia Farinha, António Francisco Ambrósio, Rufino Silva, Rosa Fernandes

**Affiliations:** ^1^Coimbra Institute for Clinical and Biomedical Research, Faculty of Medicine, University of Coimbra, Coimbra, Portugal; ^2^Center for Innovative Biomedicine and Biotechnology, University of Coimbra, Coimbra, Portugal; ^3^Coimbra University Hospital, Coimbra, Portugal; ^4^Association for Innovation and Biomedical Research on Light and Image, Coimbra, Portugal; ^5^Center for Neuroscience and Cell Biology, University of Coimbra, Coimbra, Portugal; ^6^Clinical Academic Center of Coimbra, Coimbra, Portugal

**Keywords:** diabetic retinopathy, tear fluid, inflammatory cytokines, metalloproteinases, proteome, exosomes

## Abstract

**Purpose:**

Tear fluid biomarkers may offer a non-invasive strategy for detecting diabetic patients with increased risk of developing diabetic retinopathy (DR) or increased disease progression, thus helping both improving diagnostic accuracy and understanding the pathophysiology of the disease. Here, we assessed the tear fluid of nondiabetic individuals, diabetic patients with no DR, and diabetic patients with nonproliferative DR (NPDR) or with proliferative DR (PDR) to find putative biomarkers for the diagnosis and staging of DR.

**Methods:**

Tear fluid samples were collected using Schirmer test strips from a cohort with 12 controls and 54 Type 2 Diabetes (T2D) patients, and then analyzed using mass spectrometry (MS)-based shotgun proteomics and bead-based multiplex assay. Tear fluid-derived small extracellular vesicles (EVs) were analyzed by transmission electron microscopy, Western Blotting, and nano tracking.

**Results:**

Proteomics analysis revealed that among the 682 reliably quantified proteins in tear fluid, 42 and 26 were differentially expressed in NPDR and PDR, respectively, comparing to the control group. Data are available via ProteomeXchange with identifier PXD033101. By multicomparison analyses, we also found significant changes in 32 proteins. Gene ontology (GO) annotations showed that most of these proteins are associated with oxidative stress and small EVs. Indeed, we also found that tear fluid is particularly enriched in small EVs. T2D patients with NPDR have higher IL-2/-5/-18, TNF, MMP-2/-3/-9 concentrations than the controls. In the PDR group, IL-5/-18 and MMP-3/-9 concentrations were significantly higher, whereas IL-13 was lower, compared to the controls.

**Conclusions:**

Overall, the results show alterations in tear fluid proteins profile in diabetic patients with retinopathy. Promising candidate biomarkers identified need to be validated in a large sample cohort.

## Introduction

Diabetic retinopathy (DR) is the most common complication of diabetes (1). It is the leading cause of significant vision loss and blindness in the working-age population, with significant socio-economic and quality-of-life implications (1–4). DR incidence increases with the duration of Type 2 Diabetes (T2D), the most common form of diabetes, and within 20 years of diagnosis, almost two thirds of people with T2D will have some degree of retinopathy. Based on the typical retinal microvascular lesions that become fundoscopically detectable, DR can be diagnosed and classified into two main classes: nonproliferative (NPDR) and proliferative (PDR) (5). DR is a progressive pathology, with a dynamic and varied nature from individual to individual, characterized by a set of complex changes in several key signaling pathways that coordinate communication between different retinal cells (6–8). Regular follow-up of diabetic patients could result in early detection and treatment of vision-threatening DR, enabling the prevention of up 98% of vision impairment to this condition (9). However, due to a lack of reliable markers, its diagnosis in asymptomatic patients is insufficient.

Besides neurovascular changes in the retina, a high percentage of diabetic patients develop complications in the anterior segment of the eye, including dry eye syndrome, corneal erosion, and impaired wound healing of the cornea (10). Diabetic patients can have decreased corneal sensitivity and decreased tear function. It has been reported that the impaired blood flow seen in DR can modulate the composition of tear fluid, suggesting that tears can reflect retinal changes even though there is no direct contacts between retina and tear fluid (11, 12). These alterations found in ocular surface are usually associated with inflammation, which can increase susceptibility to corneal infection and blindness (10, 13). The production of antimicrobial peptides (AMPs), which normally protect the ocular surface from bacterial infection and aid corneal wound healing by acting as anti-inflammatory mediators, is altered in tears from DR patients (13). Patients with PDR are more susceptible to impaired tear functions (14). A growing body of evidence links inflammation with diabetes-associated retinal perturbations. Upregulation of various proinflammatory cytokines, including TNF, has been reported in the vitreous/aqueous samples of diabetic patients with retinopathy (15–17). It seems that chronic inflammatory processes may also occur at the ocular surface of diabetic patients. Dysregulated inflammatory cytokine levels (MCP-1, IP-10, TNF) and decreased ratios of antiangiogenic and angiogenic cytokines were reported in T2D patients without or with DR (18, 19). Chronic hyperglycemia also triggers ocular surface changes (10). Several studies have found evidence of proteome changes in tear fluid of DR patients (12, 20, 21). Increased levels of lysozyme and low levels of lipocalin in the tear fluid were found in patients with DR (22). On the onset of PDR, lower amounts of lactoferrin and lipocalin, as well as a decrease in tear film function, were reported (22). It was also reported that diabetic and DR patients have increased levels of apolipoprotein A-1 (22). However, these studies did not assess the progression of DR or the assessment was made in a pool of tear samples prepared with different amounts of each sample (19). Therefore, tears may provide important information, namely biomarkers such as AMPs and inflammatory mediators, with clinical relevance for the diagnosis, staging and monitoring of DR.

To the best of our knowledge, a detailed examination of changes in the proteins, including AMPs, and inflammatory mediators from ocular surface of patients with diabetes and their relationship to DR has not been performed to date. In the present study, we investigated the impact of chronic hyperglycemia on ocular surface AMPs and inflammation-related proteins present in the tear fluid in diabetic humans with NPDR and PDR.

## Materials and Methods

### Patient Enrollment

This cross-sectional, non-interventional study was approved by the Ethics Committee of Centro Hospitalar e Universitário de Coimbra (CHUC; Coimbra, Portugal), with the identification CHUC-059-18. An informed consent from all participants was obtained after a detailed description of the aim and design of the study, as well as the possible complications. All procedures used in this study adhere to the tenets of the Declaration of Helsinki.

Patients with T2D, without or with DR (NPDR or PDR), as well as healthy subjects (control group) aged 40-75 years-old were included in this study. The exclusion criteria were as follows: cataract, glaucoma, or other eye diseases compromising visual acuity; systemic diseases potentially associated with tears abnormalities including rheumatoid arthritis, lupus, Sjögren's syndrome, thyroid related disorders such as Grave's disease, Hashimoto's and thyroiditis, asthma, allergies; use of anti-inflammatory, anti-bacterial or immunomodulatory drugs in the last 3 months; previous punctal plug; ocular surgery or trauma; active ocular infections or inflammations; use of contact lens within the previous 3 months; eyelid problems such as entropion, ectropion, Meibomian gland dysfunction and other anterior segment disorders and, kerato-refractive procedures (LASIK, LASEK, PRK) in the last year.

Fifty-four T2D patients with more than 5 years of diabetes duration and, under insulin therapy and/or other oral antidiabetic agents, were included in the study: 13 patients without DR, 25 patients with NPDR, and 16 patients with PDR. Twelve healthy subjects were in the control group. All criteria-satisfying control and patient groups underwent an ophthalmic examination. This ensured that inclusion and exclusion criteria were met and confirmed the stage of DR. We collected data and tear samples from both eyes of each subject whenever possible.

### Schirmer Test Type 1 and Tear Sample Collection

To measure total tear secretion and collect tear samples, a Schirmer test type 1, using Schirmer filter paper strips (Dina strip Schirmer-Plus, Dina-Hitex, Bucovice, Czech Republic), was performed. From each eye, sterile Schirmer filter paper strips were placed without anesthetic at the junction of the lateral and middle thirds of the lower eyelid and kept in place for 5 min, while subjects closed their eyes. The wet length of Schirmer strips (in mm) was registered. After tears collection, the wet portion of the strip was immediately soaked in 200 μl of 0.9% NaCl for 1 h to elute tear proteins, as previously reported (23). Eluted protein fractions were aliquoted and frozen at −80°C until analysis. Total tear protein concentration of each sample was determined by the Pierce BCA Protein Assay kit (Pierce Biotechnology Inc.), using bovine serum albumin as standard.

### Tear Film Breakup Time

TBUT test was assessed for tear film stability. Fluorescein (0.5%) was instilled into participant's tear film and the interval between instillation and appearance of the first dry spots on the cornea was measured using a broad beam of slit lamp with a blue filter. A TBUT of <10 s was considered abnormal, indicative of tear instability.

### Proteomics Analysis

#### Sample Preparation

Protein levels changes in tear samples were evaluated using mass spectrometry (MS)-based shotgun proteomics. A total of 32 samples was prepared for LC-MS/MS analyses. Proteins were denatured by addition of urea to a final concentration of 8 M in 20 mM HEPES, reduced by addition of 15 mM dithiothreitol (DTT) and incubation for 30 min at 55°C. Then proteins were alkylated by addition of 30 mM iodoacetamide (IAA) for 15 min at room temperature in the dark. Samples were diluted with 20 mM HEPES pH 8.0 to a final urea concentration of 4 M and proteins were digested with 1 μg lysyl endopeptidase (Wako) (1/100, w/w) for 4 h at 37°C. Samples were again diluted to 2 M urea and digested with 1 μg trypsin (Promega) (1/100, w/w) overnight at 37°C. The resulting peptide mixture was acidified by the addition of 1% trifluoroacetic acid (TFA) and desalted on a reversed phase (RP) C18 OMIX tip (Agilent). The tip was first washed 3 times with 100 μl pre-wash buffer [0.1% trifluoroacetic acid (TFA) in water/acetonitrile (ACN, 20:80, v/v)] and pre-equilibrated 5 times with 100 μl of washing buffer (0.1% TFA in water) before loading the sample on the tip. After peptide binding, the tip was washed 3 times with 100 μl of wash buffer and peptides were eluted twice with 100 μl elution buffer [0.1% TFA in water/ACN (40:60, v/v)]. The combined elutions were dried in a vacuum concentrator.

#### LC-MS/MS Analysis

Peptides were re-dissolved in 20 μl loading solvent A [0.1% TFA in water/ACN (98:2, v/v)] of which 2 μg were injected for LC-MS/MS analysis on an Ultimate 3000 RSLCnano system in-line connected to a Q Exactive HF mass spectrometer (Thermo). Trapping was performed at 10 μl/min for 4 min in loading solvent A on a 20 mm trapping column [made in-house, 100 μm internal diameter (I.D.), 5 μm beads, C18 Reprosil-HD, Dr. Maisch, Germany]. The peptides were separated on a 250 mm Waters nanoEase M/Z HSS T3 Column, 100Å, 1.8 μm, 75 μm inner diameter (Waters Corporation) kept at a constant temperature of 45°C. Peptides were eluted by a non-linear gradient starting at 1% MS solvent B [0.1% formic acid (FA) in water/ACN (2:8, v/v)] reaching 33% MS solvent B in 63 min, 55% MS solvent B in 87 min, 99% MS solvent B in 90 min followed by a 10-min wash at 99% MS solvent B and re-equilibration with MS solvent A (0.1% FA in water). The mass spectrometer was operated in data-dependent mode, automatically switching between MS and MS/MS acquisition for the 16 most abundant ion peaks per MS spectrum. Full-scan MS spectra (375-1,500 m/z) were acquired at a resolution of 60,000 in the Orbitrap analyzer after accumulation to a target value of 3,000,000. The 16 most intense ions above a threshold value of 15,000 were isolated with a width of 1.5 m/z for fragmentation at a normalized collision energy of 28% after filling the trap at a target value of 100,000 for maximum 50 ms. MS/MS spectra (200-2,000 m/z) were acquired at a resolution of 15,000 in the Orbitrap analyzer.

#### Data Analysis

Analysis of the mass spectrometry data was performed with MaxQuant (version 1.6.11.0) with mainly default search settings including a false discovery rate set at 1% on PSM, peptide and protein level. Spectra were searched against the human proteins in the Reference proteins database (database release version of January 2020 containing 20,365 human protein sequences, downloaded from http://www.uniprot.org). The mass tolerance for precursor and fragment ions was set to 4.5 and 20 ppm, respectively, during the main search. Enzyme specificity was set as C-terminal to arginine and lysine, also allowing cleavage at proline bonds with a maximum of two missed cleavages. Variable modifications were set to oxidation of methionine residues, acetylation of protein N-termini. Matching between runs was enabled with a matching time window of 0.7 min and an alignment time window of 20 min. Only proteins with at least one unique or razor peptide were retained. Proteins were quantified by the MaxLFQ algorithm integrated in the MaxQuant software. A minimum ratio count of two unique or razor peptides was required for quantification. A total of 312,428 peptide-to-spectrum matches (PSMs) were performed, resulting in 9,707 identified unique peptides, corresponding to 1,407 identified proteins. Further data analysis of the results was performed with the Perseus software (version 1.6.2.1) after loading the protein groups file from MaxQuant. Reverse database hits were removed, LFQ intensities were log2 transformed and replicate samples were grouped. Proteins with less than three valid values in at least one group were removed and missing values were imputed from a normal distribution around the detection limit leading to a list of 682 quantified proteins that was used for further data analysis.

### Multiplex Analyses of Matrix Metalloproteinases and Cytokines in Tears

A set of three matrix metalloproteinases (MMP-2, MMP-3 and MMP-9) and eleven cytokines (GM-CSF, IFNγ, TNF, IL-1β, IL-2, IL-4, IL-5 and IL-6, IL-12p70, IL-13, IL-18,) were analyzed using a preconfigured panel ProcartaPlex Human MMP Panel II 3plex (Thermo Fisher Scientific, Vienna, Austria) and ProcartaPlex Human TH1 TH2 11plex (Thermo Fisher Scientific), respectively. These analyses were performed using xMAP-based technology (Luminex) at the Laboratory of Immunology and Oncology (CNC-UC) and at the Blood and Transplantation Center of Coimbra (IPST). After thawing, tear samples were mixed firstly by vortex and then centrifuged at 10,000xg for 10 min, to remove eventual particulates. After, the supernatants were transferred to new Eppendorf microcentrifuge tubes. The assay workflow was performed according to the manufacturer's manual. Briefly, after 120 min incubation at room temperature of the magnetic beads with standards or samples, the wells were incubated for 30 min with detection antibody mixture and subsequently 30 min with streptavidin bound phycoerythrine solution. Between incubation times, thorough washing steps were performed. After adding reading buffer, the beads were analyzed with the Luminex instrument. Standard curves were generated by using the reference cytokine samples supplied by the manufacturer. Raw data were analyzed by ProcartaPlex Analyst 1.0 Software to obtain analyte concentrations in tear samples.

### Isolation of Small Extracellular Vesicles From Tear Fluid

Small EVs were isolated using the total Exosome Isolation Reagent (Invitrogen, Thermo Fisher Scientific, Vilnius, Lithuania), according to the manufacturer. Briefly, after an overnight incubation at 4°C of the mixture of tear fluid with isolation reagent (2:1), samples were centrifuged at 10,000xg at 4°C for 1 h. The pellet was suspended in 50 μl of filtered phosphate-buffered saline (PBS) or 150 mM NaCl, 50 mM Tris (pH 7.5), 5 mM ethylene glycol tetraacetic acid, 1% Triton X-100 (Tx-100), 0.5% sodium deoxycholate and 0.1% sodium dodecyl sulphate (SDS), supplemented with 1× protease inhibitor cocktail (Roche, Indianapolis, IN, USA), 2 mM of phenylmethylsulfonyl fluoride and 2 mM of iodoacetamide (IAD) for Nanoparticle Tracking Analysis (NTA) or Western Blotting, respectively.

### Nanoparticle Tracking Analysis

Small EVs were analyzed by performing NTA using a NanoSight NS300 instrument (Malvern Panalytical Limited, Malvern, UK). NTA acquisition settings were optimized, and the videos were used to perform the analysis and estimate the mean size and modal size and concentration of particles. Data were processed using NTA 3.3 analytical software (Malvern Panalytical Limited, Malvern, UK).

### Transmission Electron Microscopy

An aliquot of small EVs resuspended in PBS were fixed with 2% paraformaldehyde (PFA) for TEM. After deposition of PFA-fixed small EVs on Formvar-carbon coated grids (TAAB Laboratories Equipment, Berkshire, UK), grids were contrasted with uranyl acetate for 5 min. Observations were carried out under TECNAI G2 Spirit BioTWIN electron microscope (FEI) at 100 kV.

### Western Blot Analysis

Tear samples or small EVs were denatured with Laemmli buffer 5x without reducing agents (125 mM Tris-HCl (pH 6.8), 5% SDS, 20% glycerol and 0.01% bromophenol blue). Samples were loaded on polyacrylamide gels and proteins were separated by SDS-PAGE and transferred to polyvinylidene difluoride Amersham™ Hybond™ membranes (GE Healthcare, Cleveland, Ohio, USA). The membranes were blocked in 5% (m/v) nonfat milk in TBS-T (20 mM Tris, 150 mM NaCl, Tween 0.2%, pH 7.6) and probed with antibody against exosome markers CD63 (1:500; SICGEN, Cantanhede, Portugal) and flotillin-1 (1:250; Santa Cruz Biotechnology, Dallas, Texas) overnight at 4°C. After washing, the membranes were incubated with secondary anti-goat IgG-HRP-linked antibody (1:10,000; Bio-Rad, Hercules, California, USA) or anti-mouse IgG-HRP-linked antibody (1:10,000; Bio-Rad). The immunoreactive bands were detected by enhanced chemiluminescence (ECL) substrate using an imaging system (LAS500, GE Health Life Sciences, Chicago, Illinois, USA).

### Bioinformatics and Statistical Analysis

Using the Gene Ontology (GO) knowledgebase (http://geneontology.org/), a database that until 2022-01-13 had 43,786 GO terms, 7,965,896 annotations, 1,566,018 gene products, 5,128 species, a GO enrichment analysis with the 682 quantified proteins as input list was carried out. Through the database, the input list was connected to an analyzing tool of the PANTHER Classification System. A PANTHER Overrepresentation Test (Released at 2021-02-24) with Homo sapiens as reference list was performed and molecular, biological processes and cellular components annotations data set were available considering Fisher's Exact test with FDR correction (FDR *p* < 0.05). From a reference list containing 20,595 protein IDs, a total of 624 proteins were mapped, remaining 38 proteins unmapped (24, 25). Protein-protein interactome analysis was performed using STRING (https:// string-db.org, version 11.5), a database that currently covers 24,584,628 proteins from 5,090 organisms (26–37). The analysis considered both functional and physical protein associations with 0.700 (high confidence) as minimum required interaction score. Before statistical analysis, MS data were log2 transformed.

### Statistical Analysis

GraphPad Prism version 8.00 was used to perform data analysis. A *t*-test was performed (FDR = 0.05 and s0 = 1) to compare tear samples between control group and T2D, NPDR and PDR groups, and between NDPR and PDR groups, and a volcano plot was generated. For multiple comparison analysis, parameters were checked for normal distribution, given a *p* < 0.05 of the Shapiro-Wilk test. For normal distribution, one-way ANOVA followed by *post-hoc* analysis (Tukey test) was carried out to test for significance for a specific protein or analyte. Whenever a variable did not reach the normality assumption the non-parametric Kruskal-Wallis test followed by Dunn-Sidak test *post-hoc* test was performed. Differences between groups were considered significant at *p* < 0.05. The diagnostic power of biomarkers was evaluated with receiver operating characteristics (ROC) curves (AUC, confidence interval).

## Results

### Characterization of the Study Population

**Figure 1:**
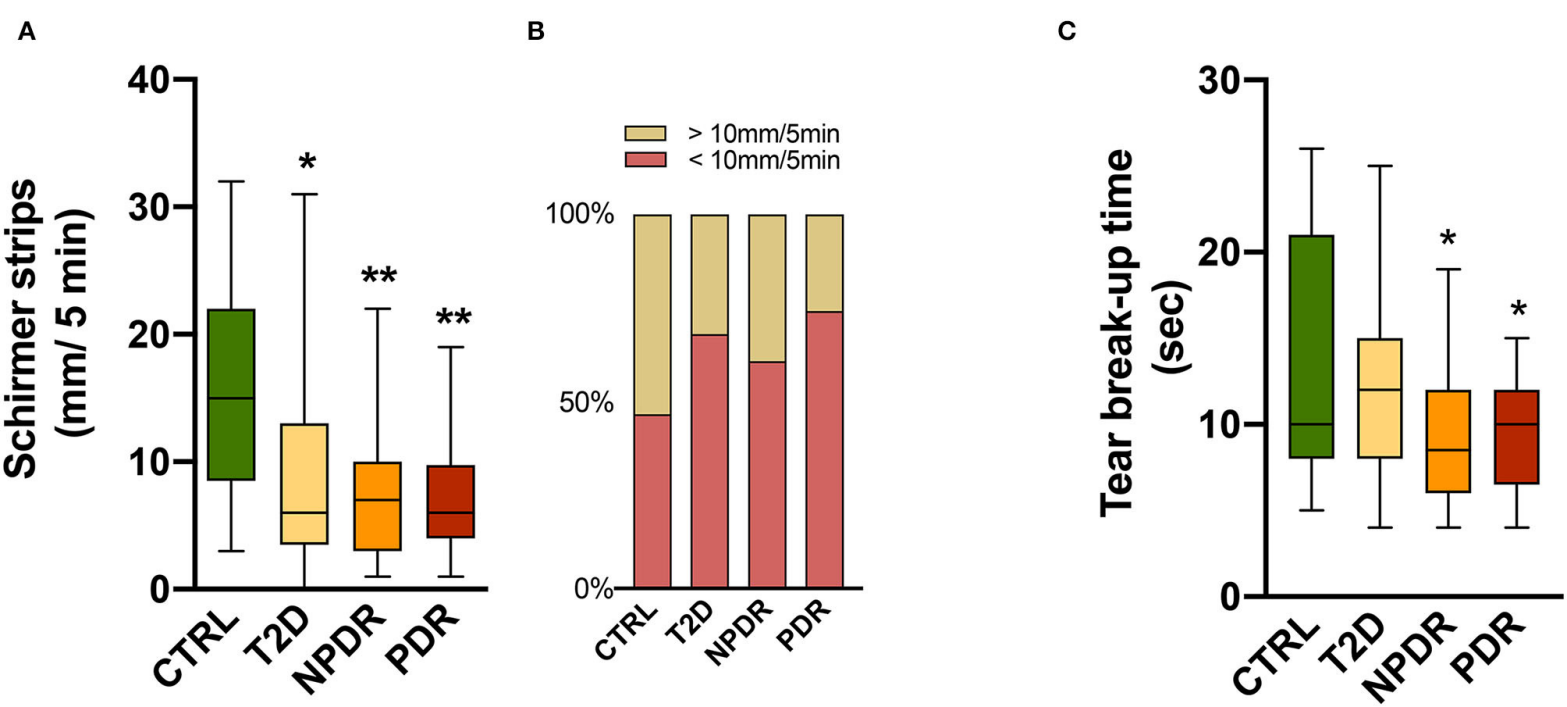
**(A,B)** Tear function tests in healthy, nondiabetic controls and T2D patients without DR or with NPDR or PDR. **(A)** Schirmer test I showing significantly reduced scores in diabetics compared to controls (Kruskal-Wallis test). **(B)** Above 60% of diabetics have <10 mm/5 min of tears volume, suggesting impairment of tears secretion or production. **(C)** Tear break-up time (TBUT) showing significantly decreased (One-way ANOVA) values in NPDR and PDR subjects, reflecting changes in tear stability in DR. Values are expressed as mean ± S.D. ^*^p < 0.05, ^**^p < 0.001 vs. CTRL. CTRL, healthy, nondiabetic control; T2D, type 2 diabetes; NPDR, nonproliferative DR; PDR, proliferative DR.

The subjects enrolled in this study were 42-75 years old in the nondiabetic control group and 40-75 years old in the T2D group, with an average age of 62 years for the entire study population. Out of 66 participants, 42 (62.1 %) were males and 24 (37.9 %) were females. Between them, 12 were healthy, nondiabetic controls (control group) and 54 T2D patients, with 13 having no DR, 25 having NPDR, and 16 having PDR (Table 1). To investigate if tear secretion and tear stability were altered, we performed Schirmer's I test and TBUT, respectively. In T2D, the Schirmer I test values reduced significantly (*p* < 0.05), with 68% of diabetic individuals having values < 10 mm/5 min. Also, 61% of T2D with NPDR and 74% with PDR had a Schirmer test < 10 mm/5 min, with Schirmer values also significantly reduced compared to the control group (*p* < 0.001) (Figures 1A,B).

**Table 1:** Clinical characteristics in control subjects (CTRL), T2D patients without DR, T2D patients with NPDR and T2D patients with PDR.

**Variables**	**Control (*n* = 12)**	**Diabetic (*****n*** **=** **54)**		
	**Healthy subjects**	**Without DR (*n* = 13)**	**NPDR (*n* = 25)**	**PDR (*n* = 16)**
Age (years)	54 ± 11	59 ± 11	65 ± 9^*^	66 ± 6
Gender (male/female)	3/9	7/6	18/7	13/3
Diabetes duration (years mean ± SD)	not applicable	12 ± 7.5	19 ± 9.2^#^	22 ± 8.7^##^
**Diabetes treatment type**
Oral medication	not applicable	82%	30%	17%
Insulin	not applicable	9%	35%	25%
Oral medication + insulin	not applicable	9%	35%	58%
HbA1c values (mean ± SEM)	-	7.1 ± 0.3	7.6 ± 0.2	7.9 ± 0.3

* *p < 0.05 vs CTRL*,

# *p < 0.05 vs Diabetic without DR*,

## *p < 0.001 vs Diabetic without DR*.

Compared with the control group, TBUT values in the NPDR and PDR groups were significantly decreased (*p* < 0.05) (Figure 1C). The results of the TBUT test indicate that individuals with DR (NPDR and PDR) have an average value below 10 s, reflecting changes in tear stability compared to controls.

### Comprehensive Global Proteome Profiling of Tear Fluid

To investigate whether tear fluid proteomic composition in DR is altered, we performed LC-MS/MS of 8 samples/group. Spectra against human protein sequences were searched in the Swiss-Prot database after LC-MS/MS runs. In all 32 samples, 312,428 peptide-to-spectrum matches (PSMs), 9,707 peptides, and 1,407 protein groups were identified in the tear fluid with a FDR at 1% at the protein and peptide spectrum match levels (Supplementary Table S1), and a total of 682 protein groups were reliably quantified (Supplementary Table S2). We checked for well-known tear markers such lipocalin-1, serum albumin, lysozyme, lactotransferrin and lactoperoxidase to guarantee the quality of our samples.

The number of proteins in each tear sample was then determined. Although there was a trend toward an increased number of proteins in tears from T2D patients with DR compared to nondiabetic control group, there were no significant differences (Supplementary Figure S1).

**Figure 2:**
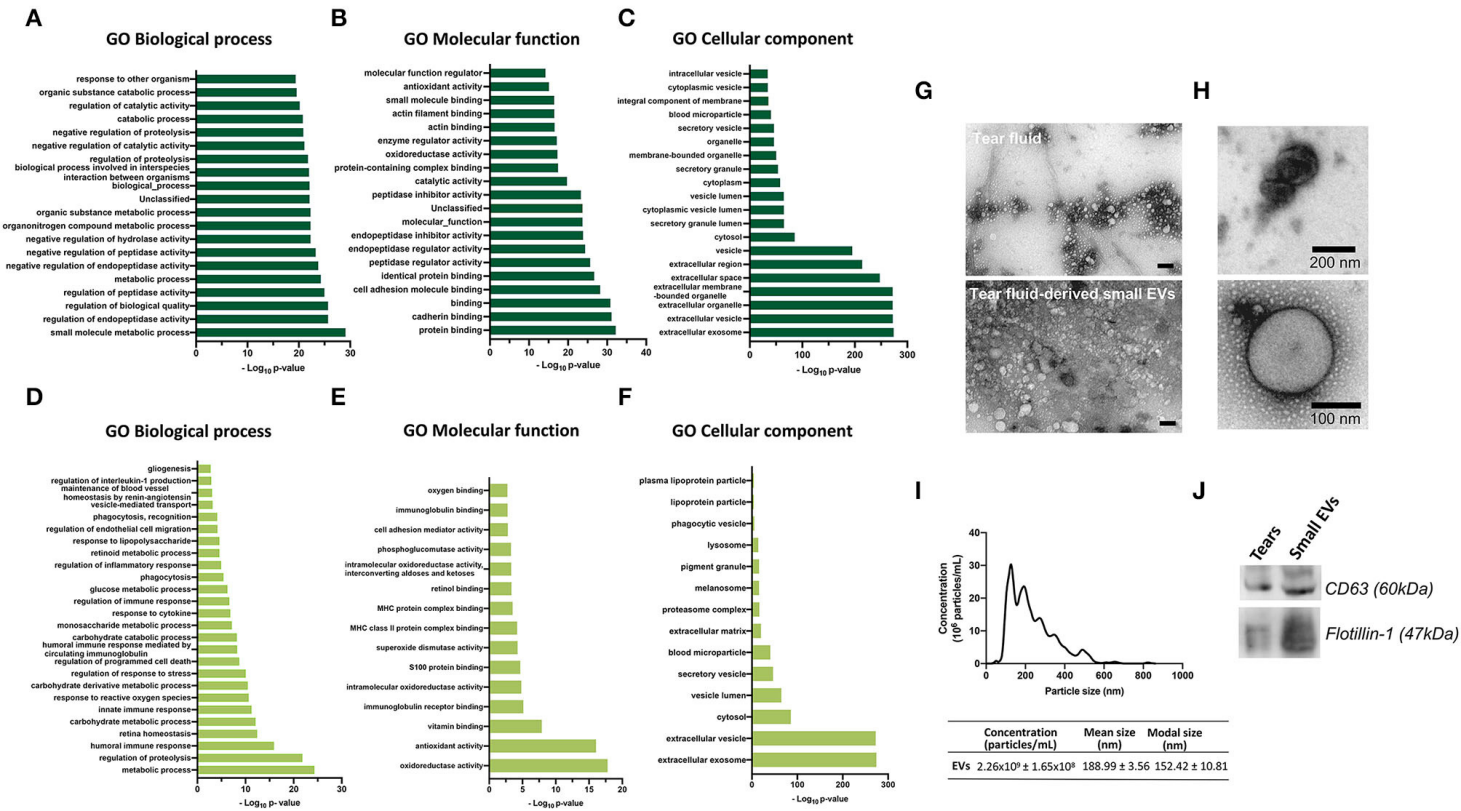
**(A–F)** Gene Ontology enrichment analysis for GO biological process, molecular function and cellular component terms. Each diagram displays the bar plot of GO category terms along the y-axis and the corresponding degree of enrichment by the –log_10_ (*p*-value) on x-axis. Top-ranked 20 enriched significant **(A)** Biological processes, **(B)** Molecular functions and **(C)** Cellular components of quantified proteins by LC-MS/MS. **(D–F)** Enriched significant biological processes, molecular functions and cellular components of 682 quantified proteins by LC-MS/MS that might be associated with the retina. [Fischer's exact test (FDR *p* < 0.05)]. **(G–J)** Tear fluid and tear-derived small EVs from healthy, nondiabetic subjects. **(G,H)** Transmission electron microscopy images showing small EVs. Small EVs were obtained using Total Exosome Isolation Reagent. Scale bar of panel G: 100 nm. **(I)** Nanoparticle tracking analysis, showing the small EVs size distributions, mean size, modal size and concentration of particles. **(J)** Western blot analysis of small EVs using antibodies against CD63 and Flotilin-1.

To analyze what biological processes, molecular functions and cellular components got overrepresented, GO enrichment analysis was performed with the quantified proteins (Figures 2A–F; Supplementary Tables S3A–C), using bioinformatics tools of the PANTHER Classification System. From a reference list containing 20,595 protein IDs, 624 proteins were identified from a list created with 682 proteins, remaining 58 unidentified. Biological process analysis revealed that “small molecule metabolic process” and “regulation of biological activity” were the most significant terms. For molecular functions, proteins were mainly enriched in “protein binding” and “cadherin binding.” The cellular components most populated were “extracellular exosome” and “extracellular vesicle” (Figures 2A–C).

Regardless of the statistical degree of enrichment analysis, for each of the GO categories, a representation of GO terms that were considered related or relevant to the physiology and/or pathogenesis of diabetes and/or DR was made. This analysis of enriched significant Biological processes, Molecular functions and Cellular components of 682 quantified proteins, revealed that highly expressed proteins might be related to response to glucose and to the retina, being involved in retina homeostasis, gliogenesis, retinoic metabolic process, regulation of endothelial cell migration and maintenance of blood vessels homeostasis by renin-angiotensin (Figures 2D–F). Moreover, the highly expressed proteins were mainly associated with regulation of proteolysis, innate and humoral immune responses, oxidative stress, and response to cytokine, among others (Figures 2D–F).

Since the cellular components were most populated with exosomal proteins, we next assessed the presence of small EVs in the tear fluid. Transmission electron microscopy of tear fluid from nondiabetic healthy controls revealed the presence of “non-vesicles” and “vesicles.” Tear fluid-derived small EVs were shown to be cup-shaped or spherical vesicles, surrounded by a well-defined membrane (Figures 2G,H). NTA revealed that these vesicles were present in the tear fluid in a high concentration (2.26 x 10^9^ ± 1.65 x 10^8^ particles/ml), where the modal size was 152.42 ± 10.81 nm, which is characteristic of small EVs (Figure 2I). Moreover, the exosome markers CD63 and Flotillin-1 were detectable in our samples, as revealed by Western blot analysis (Figure 2J).

### Protein Composition Changes in Tear Fluid of T2D Patients Without or With DR

**Figure 3:**
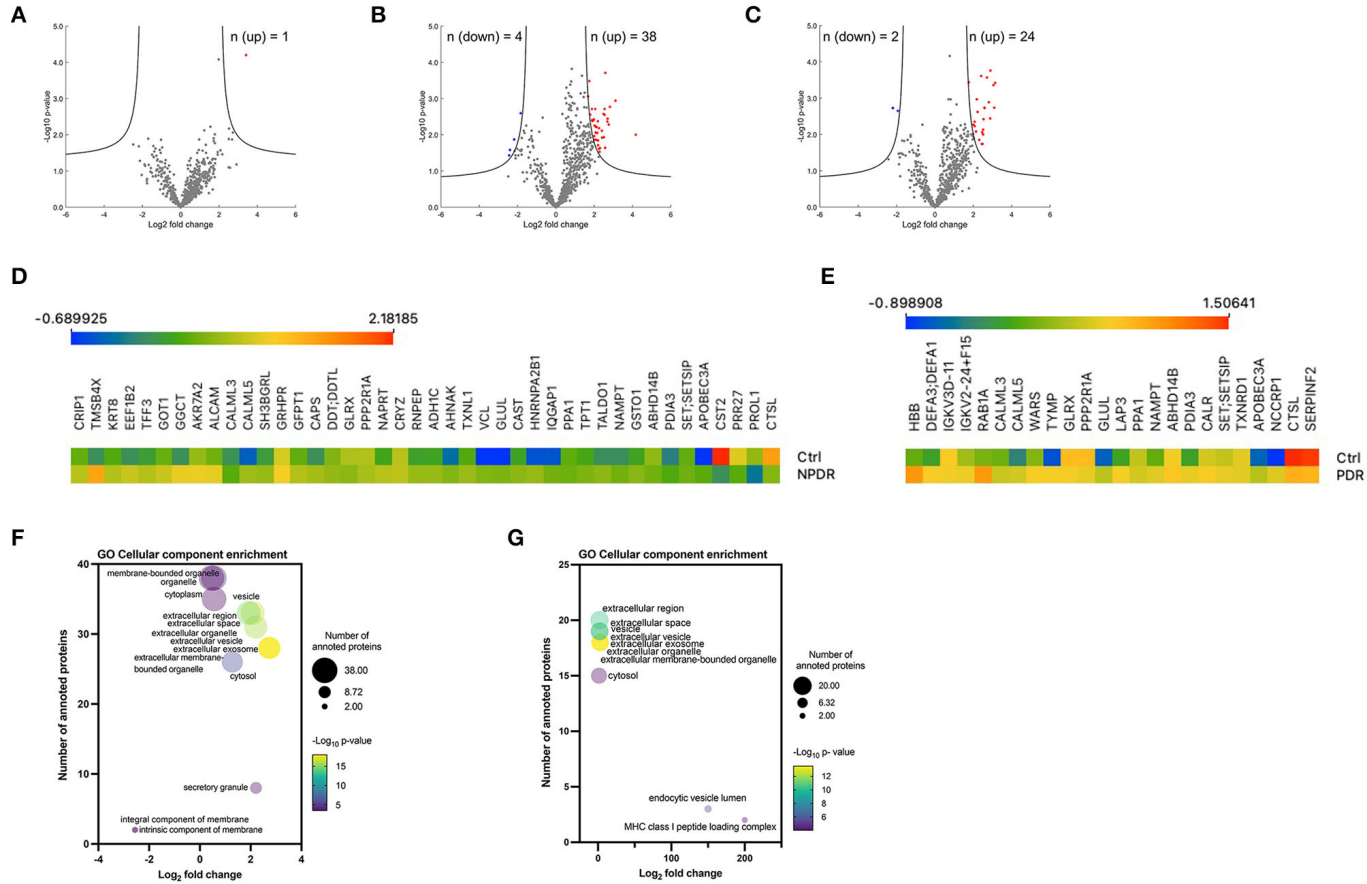
Overview of significantly regulated proteins in diabetic subgroups compared to control group. **(A–C)** Volcano plots of proteomic data. Volcano plots were generated in the software Perseus, comparing the differential protein expression in tear fluid between nondiabetic healthy controls and **(A)** T2D group, **(B)** T2D with NPDDR, and **(C)** T2D with PDR. The points indicate different proteins that display magnitude fold-changes (Log_2_; x-axis) and the *p*-values (–Log_10_; y axis) of all proteins quantified in control group and each one of the other groups (two-sample *t*-test; FDR = 0.05, S0 = 1). Proteins with significant increases are indicated by red circles. Proteins with significant decreases are depicted by blue circles. **(D,E)** Heatmaps of the differentially expressed proteins. Protein expression values were log_2_ normalized. Data corresponding to the proteins differentially expressed when comparing **(D)** NPDR and **(E)** PDR with the controls. Red indicates a high expression level; blue indicates a low expression level. **(F,G)** Bubble plots showing the enrichment for GO cellular component terms for the significantly expressed proteins in NPDR and PDR groups. The log_2_ (fold change) in x-axis represents the ratio of total proteins identified and the different proteins expected to be related to each cellular component; the size of the bubble represents the number of proteins for each cellular component and color represents the –log_10_ (*p*-value), which indicates the statistical significancy. No statistically significant enrichment was found in biological process (not presented).

To assess whether there were statistically significant differences in expression levels of each protein between the nondiabetic healthy control group and the experimental groups, a Student's *t*-test (with a correction by Benjamin and Hochberg, with FDR = 0.05 and S0 = 1) was performed. For this analysis, all the quantified proteins were considered (*n* = 682), in which a comparison was made between the samples of each group in relation to the control group. Volcano plots show the log_10_ *p*-values for each protein *vs*. the respective log_2_ fold-change. These values, as well as the statistical significance, given as a value -log p, for each protein, were plotted on a volcano graph, with fold change values shown on the X axis and –log *p*-values on the Y-axis (Figures 3A–C). Only one protein [hemoglobin subunit beta (HBB)] was upregulated in T2D tear samples, according to the volcano plot analysis (Figure 3A, Supplementary Table S4A). The volcano plot of comparison between NPDR samples and control samples, revealed 38 proteins (CALML5, EEF1B2, TXNL1, GLUL, SET/SETSIP, ALCAM, APOBEC3A, KRT8, GLRX, GGCT, AHNAK, NAMPT, VCL, SH3BGRL, ABHD14B, GRHPR, TFF3, HNRNPA2B1, PDIA3, CRIP1, DDT/DDTL, NAPRT, AKR7A2, CAPS, GSTO1, TPT1, TMSB4X, GFPT1, CRYZ, PPP2R1A, TALDO1, GOT1, CAST, IQGAP1, RNPEP, CALML3, ADH1C, PPA1) significantly downregulated (Figure 3C, Supplementary Table S4B). In PDR samples, the volcano plot showed 24 proteins (NUTF2, APOBEC3A, HBB, NCCRP1, NAMPT, SET/SETSIP, ABHD14B, DEFA3/DEFA1, GLRX, PDIA3, CAST, TYMP, GLUL, PPP2R1A, RAB1A, TXNRD1, CALML5, IGKV3D-11, IGKV2-24/IGKV2D-24, CALR, LAP3, WARS, CALML3, PPA1) significantly upregulated and 2 proteins (SERPINF2 and CTSL) significantly downregulated compared to those in control group (Figure 3C, Supplementary Table S4C). Interestingly, we did not find differences in proteins expression between the PDR and NPDR groups (Supplementary Figure S3). Heatmaps of all proteins identified to be differentially expressed are shown in Figures 3D,E. To further examine the differentially expressed proteins, GO enrichment analyses were performed. Enrichment analysis for the cellular component term, demonstrated that these proteins were mainly located in extracellular vesicles, including small EVs in NPDR group (Supplementary Table S5B, Figure 3F), or associated to MHC class I peptide loading complex and endocytic vesicle lumen in PDR group (Supplementary Table S6B, Figure 3G). In both groups, the proteins overexpressed are mainly associated with disulfide-reductase activity (Supplementary Tables S5A, S6A).

**Figure 4:**
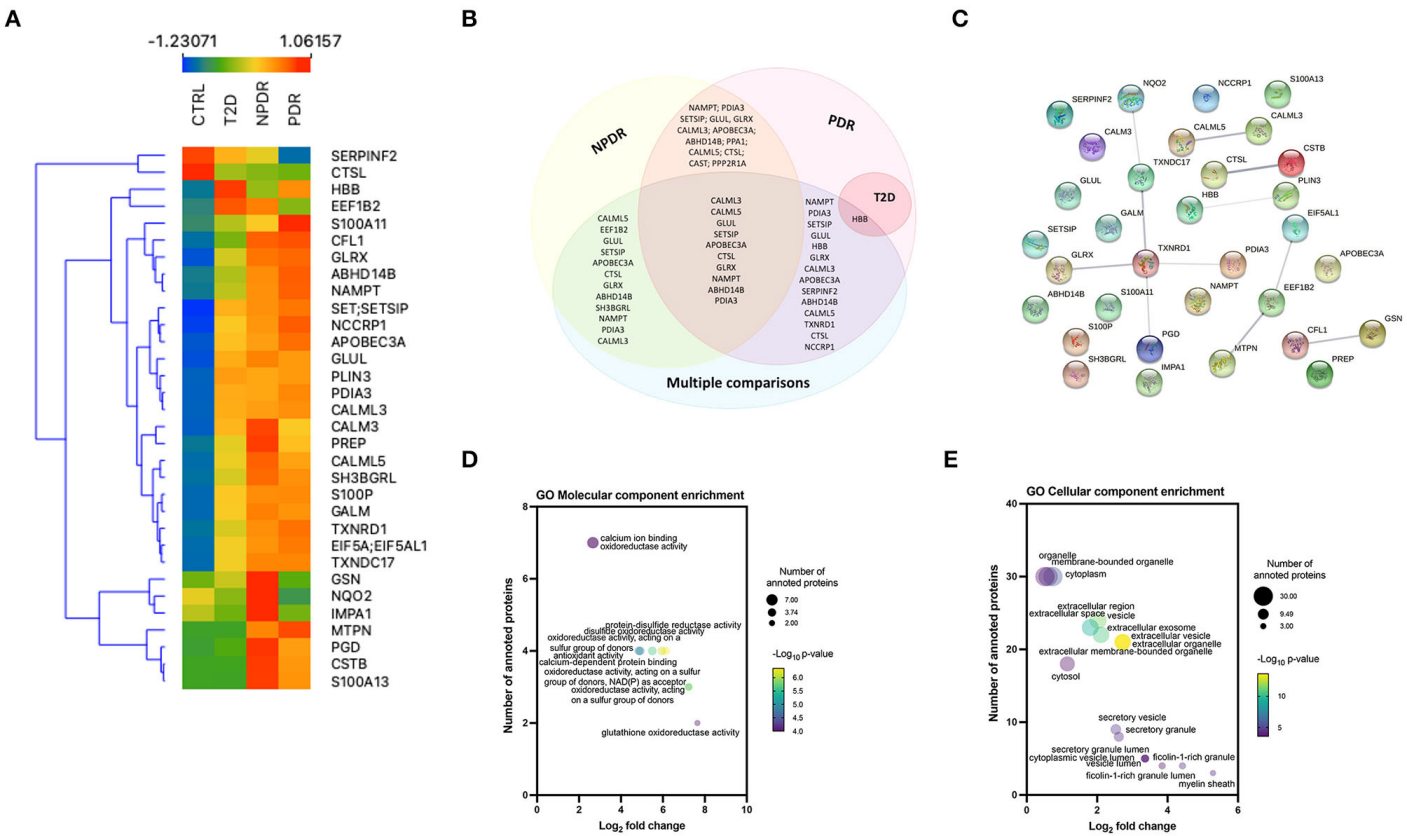
Multiple comparison analysis between the diabetic subgroups and the control group. **(A)** Heatmap of the 32 significantly expressed proteins across replicates among diabetic subgroups and control group, showing a clear distinction between all the groups, and NPDR and PDR subgroups compared to control group. **(B)** Venn diagram displaying the overlapping statistically significant proteins between each diabetic subgroup vs. control group and, the comparison of the different groups with each other (multiple comparison). **(C)** STRING network interactome analysis of statistically significant proteins in multiple comparison. PPI enrichment *p*-value: 0.00357. Each node represents the 32 statistically significant proteins, the 11 edges represent the protein-protein associations (functionally or physically) and each edge strength represents the confidence of interaction. **(D)** Bubble plot displaying the significantly enriched GO Molecular function terms for significantly expressed proteins. The log_2_ fold change in x-axis represents the ratio of proteins; the size of the bubble represents the number of proteins and color represents the statistical significancy. **(E)** Bubble plot displaying the significantly enriched GO Cellular component terms for the differentially expressed proteins in multiple comparison analysis. Extracellular vesicle and extracellular organelle represented by overlapping yellow bubbles, are the most significant cellular component. No statistically significant enrichment was obtained in biological process (data not shown).

To identify the significant differences in the proteomics profile of the different groups, we compared all the groups with each other, performing an ANOVA test (using S0 = 0 and FDR = 0.05). In this analysis, 32 proteins were significantly changed (Supplementary Figure S4; Supplementary Table S4D; Figure 4A). Nine proteins (S100A13, CSTB, SERPINF2, MTPN, GSN, PGD, NQO2, CFL1, and IMPA1) were differentially regulated in T2D patients with NPDR and PDR, compared with T2D patients without signs of DR. Significant changes in the levels of two proteins (NQO2 and IMPA1) were found in the PDR group compared with the NPDR group (Supplementary Figure S4). After differentially expressed proteins being identified between all groups, it was possible to found that 10 proteins (CALML3, CALML5, GLUL, SET/SETSIP, APOBEC3A, CTSL, GLRX, NAMPT, ABHD14B, and PDIA3) were common among NPDR and PDR, when performed a comparison between each diabetic subgroup to the control group or in multiple comparison, and 13 proteins are common to NPDR and PDR groups (Figure 4B). CALML3 and CALML5, like TXNDC17, TXNRD1, GLRX, PGD, and PDIA3, show a significant relationship, according to STRING analysis of the differentially regulated proteins (Supplementary Tables S8A,B; Figure 4C). In GO analysis of the 32 regulated proteins, we found that “glutathione oxidoreductase activity” and “regulated exocytosis” occurred significantly more frequently in the GO annotations (for molecular function and biological process, respectively) (Supplementary Tables S8C,D, Figure 4D), and “myelin sheath” and “ficolin-1-rich granule lumen” as the most enriched GO cellular components (Supplementary Table S8E, Figure 4E).

**Figure 5:**
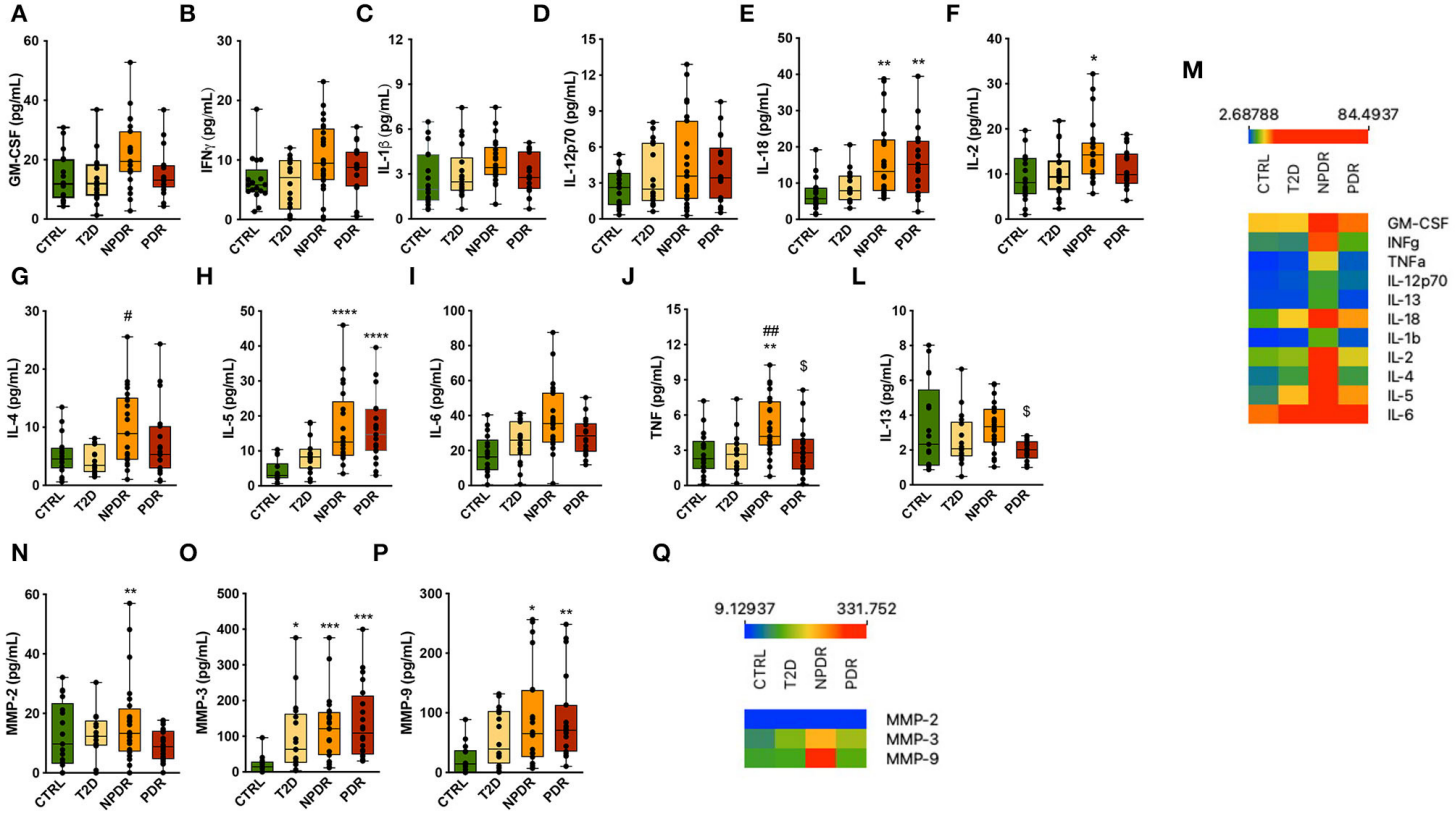
Inflammation-related mediators in nondiabetic controls and T2D patients having no DR or with NPDR or PDR. **(A–L)** Th1- and Th2-type cytokines (GM-CSF, IFNγ, IL-1β, IL-12p70, IL-18, IL-2, IL-4, IL-5, IL-6, TNF, IL-13) were measured in the tear fluid by multiplex immunoassays. The concentrations of IL-2,−4,−5,−13,−18 and TNF were significantly changed. Increased concentrations of IL-18 and IL-5 were found in NPDR and PDR groups compared to control group, and TNF and IL-2 were found to be increased only in NPDR group. IL-4 and TNF concentrations were also significantly increased in NPDR compared to T2D group. Interestingly, the concentrations of TNF and IL-13 were significantly decreased in PDR group compared to NPDR group. **(M)** Heatmap of the expression of Th1/Th2 cytokines across replicates among control group and diabetic subgroups. **(N–P)** Multiplex analysis of MMPs in tear fluid. The concentration of MMP-2 was higher in NPDR group compared to control group. Increased concentrations of MMP-3 and−9 were also found in NPDR and PDR groups. MMP-3 concentration was found to be significantly increased in T2D group. **(Q)** Heatmap of the MMP's expression across replicates among control group and diabetic subgroups. (*n* = 17-26 samples/group; Kruskal-Wallis test; ^*^p < 0.05, ^**^p < 0.01, ^***^p < 0.001 vs. CTRL. ****p <0.0001 vs. Control, #*p* <0.05 vs T2D, *##p* <0.001 vs T2D.

To understand how diabetes and DR progression affect the levels of inflammation related cytokines, we assessed the concentrations of inflammatory mediators in tears of controls and T2D patients without retinopathy and with NPDR or NPDR by multiplex immunoassays (Figure 5). We found an upregulation of pro-inflammatory cytokines, in particular IL-2, IL-18, IL-5 and TNF in NPDR group compared to nondiabetic control group (Figures 5E,F,H,J). To validate these inflammatory mediators as potential biomarkers able to differentiate NPDR group from control group, calculation of ROC was performed. High AUC revealed that IL-2 (AUC = 0.7519; CI 95% [0.5957; 0.9081]), IL-5 (AUC = 0.9218; CI 95% [0.8335; 1.000]), IL-18 (AUC = 0.8324; CI 95% [0.7024; 0.9624]) and TNF (AUC = 0.7724; CI 95% [0.6245; 0.9203]) show specificity and selectivity for NPDR group, when comparing to these tear inflammatory mediators measured in control individuals.

IL-4 and TNF were also significantly increased in this group compared to the T2D without retinopathy group (Figures 5G,J). ROC analysis revealed that IL-4 (AUC = 0.7848; CI 95% [0.6375; 0.9322] and TNF (AUC = 0.7908; CI 95% [0.6449; 0.9367]) enabled the identification of NPDR patients when compared to T2D patients without signs of DR.

Interestingly, we found that only IL-18 and IL-5 were regulated in the same way in both PDR group and NPDR groups, and for TNF and IL-13, a decrease in these cytokines was observed compared to NPDR group (Figures 5E,H,J,L,M). High AUC of IL-5 (AUC = 0.9323; CI 95% [0.853; 1.000]) and IL-18 (AUC = 0.8158; CI 95% [0.6740; 0.9575]) was obtained allowing the identification of PDR group, when compared to AUC of IL-5 and IL-18 of control group. ROC analysis revealed that IL-13 (AUC = 0.8209; CI 95% [0.684; 0.9569]) and TNF (AUC = 0.7724; CI 95% [0.6245; 0.9203]) levels predicted the classification of NPDR, when comparing to the PDR group.

MMP-2,−3 and−9 protein concentrations were also assessed. We found increased MMP-2,−3 and−9 protein levels in the tear fluid from T2D patients without or with DR compared to nondiabetic healthy controls (Figures 5N–Q). ROC analysis revealed that MMP-3 (AUC = 0.8930; CI 95% [0.7904; 0.9956] for NPDR and (AUC = 0.9500; CI 95% [0.8747; 1.000] for PDR) and MMP-9 (AUC = 0.7919; CI 95% [0.6473; 0.9366] for NPDR and (AUC = 0.8609; CI 95% [0.7306; 0.9912] for PDR) tear levels predicted the identification of NPDR and PDR groups, when comparing to the control group. MMP-2 showed a lower AUC (AUC = 0.5711; CI 95% [0.3844; 0.7577], and so cannot be considered a reliable biomarker to identify the NPDR group, when compared to the control group.

## Discussion

In recent years, the interest in tear fluid as a potential source of biomarkers to diagnose several diseases has been increasing, due to its relatively easy non-invasive access and simple composition compared to other body fluids, such as blood and serum. Besides that, a gentle collection of tears, such as the one used in this study (with Schirmer test strips), enables the assessment of tear components (proteins/AMPs and inflammatory mediators) that eventually may be associated with DR. Herein, we designed a cross sectional, non-interventional study comprising nondiabetic, healthy controls, patients with T2D without retinopathy, with NPDR and PDR to investigate whether the tear fluid can be a source of biomarkers for diagnosis of DR.

According to several studies, dry eye syndrome is more common in diabetic individuals, which might be explained by reduced tear production or secretion mainly due to autonomic nervous system dysfunction (13, 38–42). In the population studied, with an average of 62 years, we found a significant proportion of individuals with decreased tear secretion, even in the healthy group. This is consistent with the fact that tear production declines with age due to the involution of the lacrimal and Meibomian glands and nerve activities that regulate them (43). Even though we did not conduct a comprehensive study, including a McMonnies' dry eye questionnaire or other questionnaires (44–47), to validate some of the symptoms associated with dry eye, such as foreign body sensation or itching, we detected dry eye using Schirmer's test. In addition, we performed another tear function assay, the TBUT. Schirmer and TBUT values were significantly reduced in NPDR and PDR groups, meaning a dysfunction of tear fluid production and stability in DR. These results are corroborated by some reports (39, 48) which suggest that diabetic individuals are more likely to suffer from dry eye syndrome than their age-matched peers and that with the progression of diabetes and DR, the risk is even greater. A study reported that autonomic neuropathy is present in 75% of diabetic patients with PDR (49). Chronic hyperglycemia, peripheral autonomic neuropathy, reduced insulin levels, and microvascular dysfunction, are risk factors for dry eye, that in diabetic subjects cause the decreased density of neuronal fibers of lacrimal glands and cornea, modifications of the cornea and conjunctiva epithelium, and increased osmolarity of the tear film (42). Altogether, they contribute to an inflammatory environment. These results suggest that dysfunction of the lacrimal functional unit may be related to the progression to dry eye disease in T2D patients. However, we should be cautious and should not discard that most of the diabetic patients take drugs, such as beta blockers and diuretics, commonly used to treat hypertension, which can also inhibit the production of tears.

In this study, we used Schirmer test strips to collect tear samples for proteomics analysis and assessed proteins of interest by a bead-based multiplexed immunoassay. We performed label-free quantitative proteomics analysis to compare the tear fluid of nondiabetic, healthy controls with T2D diabetic patients with no retinopathy, T2D patients with NPDR or PDR. There have been a few proteomics studies on the tear fluid in DR, however, most of them did not compare the four groups as we did (20, 21, 50–55). Overall, we identified 1,407 protein groups from tear fluid, being 682 proteins reliably quantified. In general, many of these proteins have already been described to be present in tear fluid in previous proteomics studies (56–58). However, a complex analysis involving healthy, diabetic patients without retinopathy and diabetic individuals with the main stages of retinopathy, to our knowledge, had not yet been performed.

We used gene enrichment analysis to find the enrichment of expressed proteins in biological processes, cellular components, and molecular function. We noticed that the proteomics profile of our enriched results indicated that significantly high expressed proteins in tear fluid are involved in key processes for the preservation of retinal homeostasis, including regulation of endothelial cell migration (fibroblast growth factor (A0A2I2YE38), annexin (G3QPT3), fibroblast growth factor (G3QIJ1), multifunctional fusion protein (FGF1), PRCP isoform 3 (PRCP)] and maintenance of vessel diameter [(transforming protein RhoA (RHOA), angiotensinogen (AGT) and alpha-1-antiplasmin (SERPINF2)]. The retinal vascular network is arranged hierarchically and plays a key role in homeostasis and disease. Endothelial cells form a single cell layer that lines all blood vessels and can respond to a variety of biomechanical stimuli in their environment, leading them to migrate and promote vascular morphogenesis or angiogenesis (59). The retina is a complex structure with intricate anatomical connections between the microvasculature, neurons, and glia. The local renin-angiotensin system (present in retinal microvessels, macroglial Müller cells, and ganglion cells) (60) has a key role in systemic vascular control and electrolyte homeostasis, and dysfunction in this system is often associated with diseases of retinal vasculature, such as DR (61). Moreover, other proteins that are highly enriched in tear fluid are related to the processing of antigens (MHC II molecules), regulation of proteolysis, oxidative stress, and response to cytokine, which are critical modulators of innate immune responses in DR (62, 63).

Among the 682 proteins, we identified 13 proteins that were commonly changed among diabetic subjects with different stages of retinopathy, implying that the amounts of proteins in tear fluid might indicate the presence of DR. Moreover, this knowledge can be used to better understand the molecular mechanisms underlying DR and identify target candidates of the disease. In the multiple comparisons between the four groups, 32 proteins were found to be differentially expressed, with 10 of them [Calmodulin-like protein 5 (CALML5), Glutamine synthetase (GLUL), Protein SET; Protein SETSIP (SET/SETSIP), DNA dC->dU-editing enzyme APOBEC-3A (APOBEC3A), Cathepsin L1 (CTSL), Glutaredoxin-1 (GLRX), Nicotinamide phosphoribosyltransferase (NAMPT), Alpha/beta hydrolase domain-containing protein 14B (ABHD14B), Protein disulfide-isomerase A3 (PDIA3), and Calmodulin-like protein 3 (CALML3)] not known to be highly expressed in tear fluid. These immunomodulatory proteins are associated with oxidative stress response and are relevant in angiogenesis and the healing process (13). For example, the proteins Thymidine phosphorylase (TYMP), Glutamine synthetase (GLUL), Alpha-2-antiplasmin (SERPINF2), and Tryptophan–tRNA ligase (WARS1) form a cluster related to blood vessel morphogenesis and development. These proteins significantly changed in the PDR group compared to the control group. With this information, we can infer a probable implication in the pathogenesis of retinopathy. Cathepsin L1 (CTSL), Thymidine phosphorylase (TYMP), DNA dC->dU-editing enzyme APOBEC-3A (APOBEC3A) and F-box only protein 50 (NCCRP1) form a cluster related to carbohydrate derivative catabolic process, while Cathepsin L1 (CTSL), Ras-related protein Rab-1A (RAB1A) and F-box only protein 50 (NCCRP1) form a cluster related to glycoprotein metabolic process. These findings are relatively attractive because the levels of these proteins were shown to be significantly changed when a multiple comparison test was performed between the four groups, implying a relation with diabetes progression and microvascular complications such as DR. Ig kappa chain V-III region VG (IGKV3D-11) and Immunoglobulin kappa variable 2-24 (IGKV2D-24) are involved in the production of molecular mediators of immune response, immunoglobulin production and adaptive immune response processes, with the first two proteins being significantly changed only in the PDR group compared to the control group.

A previous study reported 20 proteins differently expressed in tears from diabetic individuals compared to healthy individuals by the ESI-Q-TOF MS/MS analysis (21). Among these, 2 were up-regulated (beta-2 microglobulin and DJ-1 protein) and the others were down-regulated (S100A4/A8/A9, adenine phosphoribosyl transferase isoform, envelop protein, keratin 31, SAP1 protein, lipocalin 1-like-1, lipocalin, cytokeratin 4, lipocalin 1 precursor, HSP27, beta globin phosphohistidine phosphatase, phosphohistidine phosphatase). In other studies, NPDR patients had reduced levels of lipocalin-1, HSP27, beta-microglobulin in tears and increased levels of endothelin and neuron-specific enolase, while PDR subjects had increased levels of nerve growth factor, APOA1, lipocalin 1, lactotransferrin, lacritin, lipophilin A and Immunoglobulin lambda chain (21). Some of these proteins were also identified in our study, although without statistically significant differences, probably due to the small sample size. This might limit the interpretation of the results obtained. Nevertheless, these proteins are described as being involved in immune processes, inflammatory and oxidative stress processes. Although they are described as abundant proteins in the tear fluid, it is not yet known if they have a direct implication in DR pathophysiology. However, certain aspects such as the type of study and groups involved, as well as, the type of tears collected, the extraction procedures, and the proteomics techniques chosen for each type of study, must be considered in this comparative analysis between the present study and those previous studies. Furthermore, before being translated into clinical practice, proteomic findings must be confirmed using a larger cohort of samples and other methodologies.

Currently, there are a few promising circulating biomarkers for which verification evidence is now available (64). HbA1c levels, for example, have been shown to be a good predictor of DR risk and a useful clinical indicator when combined with other markers (65). Besides HbA1c, other protein biomarkers identified in circulation, saliva, vitreous or tears include basement membrane and extracellular matrix turnover markers [Collagen IV, Matrix metalloproteinases (MMPs)], enzyme inhibitors [cystatin C, α-2-macroglobulin (A2MG)], plasma protein transport regulators [afamin (AFM), apolipoproteins, retinol binding protein 4 (RBP4)], coagulation cascade mediators (complement cascade proteins and serpinA4), inflammatory mediators such as lipoprotein-associated phospholipase A2 (Lp-PLA2), leucine-rich alpha-2-glycoprotein (LRG1), Interleukin-6, TNF, and other circulating factors such as advanced glycation end products and vascular endothelial growth factor (VEGF). Some of them were reported to be elevated in early and intermediate phases of NPDR (for example, IL-6, VEGF, and AGES) (64). Serum levels of transforming growth factor β (TGF- β1) have been recently shown to be predictive of DR progression from NPDR to PDR (66). Moreover, recent evidence has shown a correlation between specific miRNA and intraretinal hyper-reflective spots, assessed by optical coherence tomography (67).

One of the enrichments analyses, the GO cellular component, showed that most of the quantified proteins are associated with the extracellular space and are present in extracellular vesicles, including exosomes. These data corroborate a study that states that proteins enriched in tear fluid (from principal and accessories lacrimal glands, as well as from cells of the ocular surface) are mostly from the extracellular region, whereas proteins from the lacrimal fluid (exclusively from lacrimal gland) are mostly cytosolic, followed by the extracellular proteins. Considering that the tears were collected using the Schirmer test, the samples contain proteins secreted not only by the tear glands but also by the epithelial cells of the ocular surface, stromal immune cells and meibomian and gland acinar cells, justifying the results obtained. Although we found that tear fluid is enriched in small EVs, we did not investigate whether their number or the levels of small EVs proteins are altered in the tear fluid of subgroups of T2D patients. Furthermore, we found the presence of structures with typical characteristics of small EVs both in the total tear fluid and in samples of isolated small EVs. Interestingly, other distinct structures were also observed. It was previously reported that exosome isolation using a precipitation-based approach, such as the one used in this work, can result in the presence of contaminating structures such as “non vesicles,” microparticles, cell debris, and macroaggregates (68). In this work, the isolation method consists of the use of the polymer polyethylene glycol, to dehydrate and precipitate the vesicles. However, besides their precipitation, other extracellular vesicles, protein aggregates, and extracellular proteins can also be concomitantly isolated. To unveil the biological information these vesicles can be conveying, and the cells implicated in their production, further studies are required to carefully test and characterize the extracellular vesicles-derived tear fluid, not just from a physical standpoint but primarily from a composition one.

Studies indicate that the inflammatory environment associated with lacrimal functional unit dysfunction, and the pathophysiological of diabetes/ DR, are mediated by changes in inflammatory mediators (7, 8, 69–73). In this study, we analyzed 11 cytokines, from which, IL-2, IL-1b, TNF, IL-12p70, GM-CSF, and IFNγ are produced primarily by human CD4+ T-helper (Th) 1 cells, and IL-4, IL-5, IL-6, IL-13, IL-12p70, and IL-18 are mainly produced by Th2 cells. Th1 and Th2 cells are linked to inflammation and hypersensitivity and enhance both cellular and humoral immune responses. Previous reports demonstrated the presence of Th1 and Th2 cytokines in vitreous samples (74). We assessed the levels of these cytokines in our study to get a better understanding of the inflammatory process in T2D with DR. We found increased concentrations of various inflammatory cytokines (IL-2,−4,−5,−18, and TNF) in T2D patients with NPDR. The diagnostic power of biomarkers evaluated with ROC curves revealed that tears IL-2 and TNF present in tears can be considered acceptable biomarkers, and IL-18 and IL-5 can be excellent biomarkers for the discrimination of NPDR patients from control individuals.

We also found increased concentrations of IL-4 and TNF in the NPDR group compared to the T2D group. In this case, IL-4 and TNF can be considered acceptable biomarkers to discriminate NPDR group from T2D patients without signs of DR. Although no statistically significant changes in GM-CSF, IFNγ, IL2p70 and IL-6, there was a trend toward an increase in their concentrations in the NPDR group compared to control group. As a result, it is plausible that a disruption of the balance of pro-inflammatory and anti-inflammatory factors, required for retinal homeostasis, underly NPDR. Activated microglia, endothelial cells, macroglia, and neurons can produce increased levels of pro-inflammatory cytokines in the early stages of DR and contribute to exacerbating inflammatory response throughout all cell types of the retina. It has been reported that these mediators, with exception of IL-18, are also increased in serum and ocular (aqueous or vitreous samples) of both diabetic patients with NPDR and PDR. Interestingly, we did not find gradual increases in concentrations with the degree of DR, and we only found significantly higher concentrations of IL-5 and IL-18 in the PDR group than in the control group. Like in the NPDR group, the two interleukins revealed to be potential biomarkers for the discrimination of PDR patients from control individuals. IL-18 has been associated with retinal degenerative diseases, playing a critical role in angiogenesis (75). In the samples of tear fluid from the PDR group, there was a down-regulation of the anti-inflammatory cytokine IL-13 compared to the NPDR group, suggesting a decrease of anti-inflammatory activity in the tears of individuals with PDR. We also found decreased levels of TNF in the PDR group compared to the NPDR group, while other authors have detected higher TNF levels in the tear fluid of the PDR group (50). To understand the association between the lowered levels of protein factors and DR, more research into the precise roles of TNF- and other cytokines in the development of DR is needed.

Based on previous findings showing the role of MMPs on pathological processes related to DR (76, 77), we analyzed MMP-2,−3, and−9 in the tear fluid of control subjects, diabetic patients without signs of DR and diabetic patients with NPDR or PDR. We found increased levels of MMP-3 and−9 in the tear fluid of diabetic individuals with DR, which were shown to be promising biomarkers of DR, having an AUC close to 1. Although MMP-2 levels were also increased in the tear fluid of the NPDR group, it did not present a high AUC value in the ROC curve, meaning that it is not a good predictive biomarker for DR. Elevated levels of MMP-2 and−9 were previously reported in the vitreous and retina in patients with DR and animal models of the disease (76, 78, 79). Moreover, they were shown to act as pro-apoptotic, accelerating the apoptosis of retinal neuronal and endothelial cells (76, 79). Additionally, they were shown to play an important role in the development of DR, more specifically, their increased levels in the diabetic retina facilitate the increase in vascular permeability, through proteolytic degradation of the tight junction complexes (76). MMPs have also been reported to act on pro-inflammatory mediators, playing an important role in the switch in acute and chronic inflammation (77). MMPs also facilitate neovascularization in the advanced stages (77).

Although inflammatory mediators were not detected in proteomics analysis, some of the proteins that were identified by proteomics as significantly differentially expressed in multiple comparisons have been described as related to inflammation in DR. For example, S100A13 was significantly upregulated in diabetic individuals with NPDR. It has been reported to be involved in angiogenesis and cell apoptosis and has a moderately strong binding to receptors for advanced glycation end products (RAGE) which are involved in inflammatory processes of diabetes (80). Another example is GLRX, which was significantly increased in both stages of retinopathy. It was previously reported an increase in GLRX in the retinas of diabetic rats and retinal Müller glial cells cultured in high glucose. GLRX regulates NF-κB activation and induction of the inflammatory mediator intercellular adhesion molecule-1 (ICAM-1) (81). In the multiplex bead immunoassay, the quantification of inflammatory mediators allowed us to verify this trend, with an increase of the pro-inflammatory cytokines IL-2, IL-18, IL-5 and TNF particularly in diabetic individuals with NPDR.

The proteomic study and multiplex immunoassay of the tears from patients with DR revealed valuable molecular information regarding already identified and novel proteins that are changed in tears in the context of this disease. After validating proteomics data using a larger cohort of individuals and a different methodological approach, a set of biomarkers can be identified and validated for DR diagnosis. These changes could also contribute to gaining a better understanding of DR.

## Conclusions

In this study, we identified several proteins in tear fluid that are changed in the context of DR. Our findings not only confirm the presence of dry eye syndrome in patients with DR, but also unveil specific protein profile changes that are not present in DR patients. The altered proteins in tear fluid are associated with various biological processes, such as oxidative stress, immune response, and inflammation, characteristic of DR. Although a major limitation of this study is the small number of samples, the information presented here offers a foundation for future research into biomarkers in tear fluid and eventually in tear fluid-derived extracellular vesicles. A study with a larger sample size should be performed to validate our results. The identification of a set of biomarkers can improve the early diagnosis of DR and ensure prompt treatment for this vision-threatening disease.

## Data Availability Statement

The mass spectrometry proteomics data have been deposited to the ProteomeXchange Consortium via the PRIDE (82) partner repository with the dataset identifier PXD033101 and 10.6019/PXD033101.

## Ethics Statement

The studies involving human participants were reviewed and approved by Comissão de Ética para a Saúde do CHUC, E.P.E. - Centro Hospitalar e Universitário de Coimbra, Praceta Prof. Mota Pinto 3000-075 Coimbra. The patients/participants provided their written informed consent to participate in this study.

## Author Contributions

EC, EL, FR, AFA, and RF: study design. GT, CG, JS, PB, IM, CF, and RS: clinical management. MA, BM, TR-R, PR-S, FC, and RF: data collection and analysis. MA, HG, and RF: data curation. MA and RF: writing—original draft preparation. HG, AFA, and RF: writing—review and editing. RS, and RF: supervision. RF: funding acquisition and project administration. All authors have read and agreed to the published version of the manuscript.

## Funding

This study was funded by Faculty of Medicine, University of Coimbra/Santander-Totta (PEPITA Program), Study Group In Fundamental and Translational Research (GIFT) of the Portuguese Society of Diabetology (SPD), Portuguese National Funding Agency for Science and Technology (FCT) and Strategic Projects UIDB/04539/2020 and UIDP/04539/2020 (CIBB), and COMPETE-FEDER (POCI-01-0145-FEDER-007440); Centro 2020 Regional Operational Program: BRAINHEALTH 2020 (CENTRO-01-0145-FEDER-000008).

## Conflict of Interest

The authors declare that the research was conducted in the absence of any commercial or financial relationships that could be construed as a potential conflict of interest.

## Publisher's Note

All claims expressed in this article are solely those of the authors and do not necessarily represent those of their affiliated organizations, or those of the publisher, the editors and the reviewers. Any product that may be evaluated in this article, or claim that may be made by its manufacturer, is not guaranteed or endorsed by the publisher.

## Abbreviations

DR: Diabetic retinopathy
NPDR: nonproliferative diabetic retinopathy
PDR: proliferative diabetic retinopathy
T2D: Type 2 Diabetes
GO: Gene ontology
AMPs: antimicrobial peptides
MMP: matrix metalloproteinase
LFQ: label-free quantification
SDS: sodium dodecyl sulphate
FDR: false discovery rate.
