# Supplementary material for: Putative Biomarkers in Tears for Diabetic Retinopathy Diagnosis
Source: Front Med (Lausanne). 2022 May 25;9:873483. doi: 10.3389/fmed.2022.873483 (PMC9174990; doi:10.3389/fmed.2022.873483)
Supplement: Supplementary file 9 [file Data_Sheet_1.docx]

Supplementary Material

**SUPPLEMENTARY INFORMATION for Amorim M. *et al***

Putative biomarkers for diabetic retinopathy diagnosis and progression

**Authors:**

Madania Amorim^1,2^, Beatriz Martins^1,2^, Francisco Caramelo^1,2^, Conceição Gonçalves^3^, Grimalde Trindade^3^, Jorge Simão^3^, Patrícia Barreto^4^, Inês Marques^4^, Ermelindo Leal^2,5^, Eugénia Carvalho^2,5^, Flávio Reis^1,2,6^, Teresa Rodrigues^1,2^, Henrique Girão^1,2,6^, Paulo Rodrigues-Santos^2^, Cláudia Farinha^3,4^, António Francisco Ambrósio^1,2,4,6^, Rufino Silva^1,2,3,4,6^ and Rosa Fernandes^1,2,4,5,6,^*

**Affiliations:**

^1^Univ Coimbra, Coimbra Institute for Clinical and Biomedical Research (iCBR), Faculty of Medicine, Coimbra, Portugal;

^2^Univ Coimbra, Center for Innovative Biomedicine and Biotechnology (CIBB), Coimbra, Portugal;

^3^Coimbra University Hospital, Coimbra, Portugal;

^4^Association for Innovation and Biomedical Research on Light and Image (AIBILI), Coimbra, Portugal;

^5^Univ Coimbra, Center for Neuroscience and Cell Biology (CNC), Portugal;

^6^Clinical Academic Center of Coimbra (CACC), Coimbra, Portugal.

*Corresponding author:

Rosa Fernandes

Coimbra Institute for Clinical and Biomedical Research (iCBR), Faculty of Medicine, University of Coimbra, Azinhaga de Santa Comba, 3000-548 Coimbra, Portugal.

Phone: +351 239480072

E-mail: rcfernandes@fmed.uc.pt

**Supplementary Figure S1.** Number of proteins present in tear fluid from nondiabetic controls (CTRL), T2D patients without DR, T2D patients with NPDR and T2D patients with PDR. N=8 samples/ group. All values are presented as mean ± SEM. (One-way ANOVA; NPDR vs CTRL, p=0.0552; PDR vs CTRL, p=0.0854).

**Supplementary Figure S2.** Un-cropped membranes of Western blot images shown in Figure 2J.


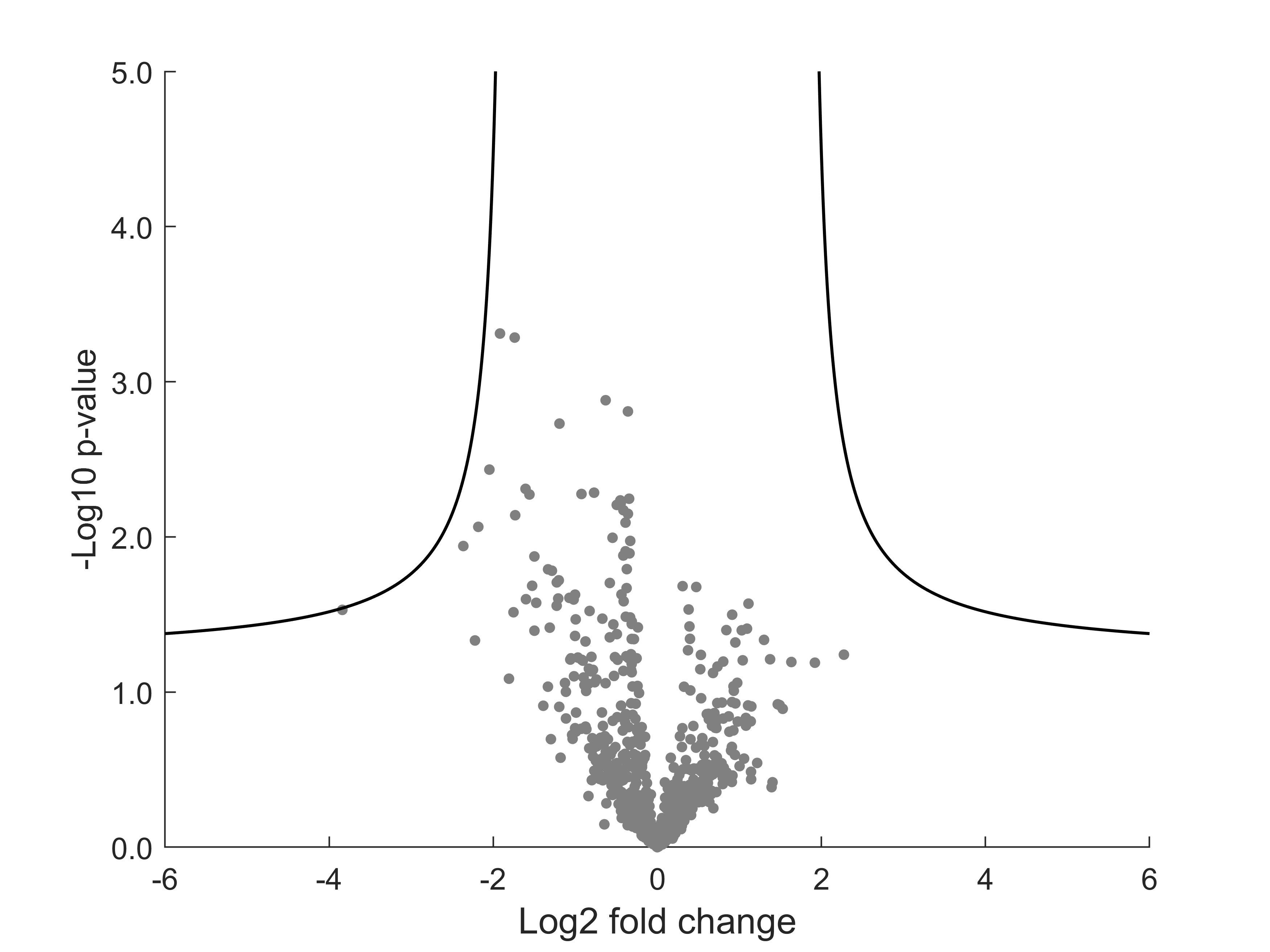


**Supplementary Figure S3.** Volcano plot of proteomic data showing no differences in the expression of proteins in the PDR group compared to the NPDR group.

**Supplementary Figure S4. Multiple comparisons of LFQ intensities of the 32 proteins.** The graphs show the comparisons of LFQ intensities between nondiabetic healthy control subjects (CTRL) and diabetic subjects without and with retinopathy (NPDR and PDR) for the 32 statistically significant proteins detected in the ANOVA test. *p<0.05, **p<0.01, ***p<0.001 vs CTRL; ^#^p<0.05, ^##^p<0.01, ^###^p<0.001 vs T2D; ^$^p<0.05, ^$$^p<0.01 vs NPDR.
